# Paternal Depression and Risk of Depression Among Offspring: A Systematic Review and Meta-Analysis

**DOI:** 10.1001/jamanetworkopen.2023.29159

**Published:** 2023-08-16

**Authors:** Berihun Dachew, Getinet Ayano, Bereket Duko, Blake Lawrence, Kim Betts, Rosa Alati

**Affiliations:** 1 School of Population Health, Faculty of Health Science, Curtin University, Perth, Western Australia, Australia; 2 enAble Institute, Curtin University, Perth, Western Australia, Australia; 3 Australian Centre for Precision Health, University of South Australia, Adelaide, South Australia, Australia; 4 South Australian Health and Medical Research Institute, Adelaide, South Australia, Australia; 5 Institute for Social Science Research, University of Queensland, Brisbane, Queensland, Australia

## Abstract

**Importance:**

Existing epidemiological evidence is equivocal as to whether paternal depression poses a consequent risk of depression in offspring; meta-analysis of findings can help inform preventative intervention efforts.

**Objective:**

To conduct a systematic review and meta-analysis of observational studies examining the association between paternal and offspring depression.

**Data Sources:**

Embase, PubMed, PsycINFO, Scopus, and Web of Science databases were searched between inception and December 2022.

**Study Selection:**

The review included all observational studies that investigated the association between paternal and offspring depression and 10 606 studies were initially identified.

**Data Extraction and Synthesis:**

This systematic review and meta-analysis was performed following the Preferred Reporting Items for Systematic Review and Meta-analysis guidelines. The review protocol was prospectively registered in PROSPERO. Summary odds ratios (ORs) and 95% CIs were pooled using inverse variance weighted random effect meta-analysis. Subgroup and sensitivity analyses were performed.

**Main Outcomes and Measures:**

The main outcome of interest was offspring depression measured using recognized depression assessment tools.

**Results:**

Sixteen observational studies published between 2002 and 2021 were included, with a combined sample of 7 153 723 father-child dyads. A meta-analysis of these studies showed that paternal depression was associated with an increased risk of depression in offspring (OR, 1.42; 95% CI, 1.17-1.71). The risk was higher among offspring exposed to paternal depressive disorders (OR, 1.65; 95% CI, 1.28-2.12) than those exposed to depression as defined by a nonclinical symptom scale (OR, 1.12; 95% CI, 1.06-1.19). Sensitivity analysis revealed consistent pooled estimates ranging from 1.35 (95% CI, 1.12-1.62) to 1.45 (95% CI, 1.18-1.78).

**Conclusions and Relevance:**

Paternal depression was associated with subsequent offspring depression. This finding shows the intergenerational transmission of mental health problems and suggests that mental health interventions benefit not only the patient but also the family as a whole, including both parents.

## Key Points

**Question:**

Is paternal depression associated with subsequent offspring depression?

**Findings:**

In this systematic review and meta-analysis including 7 153 723 father-child dyads from 16 observational studies, paternal depression was associated with a 42% increased risk of depression in offspring.

**Meaning:**

These findings suggest the importance of addressing maternal and paternal mental health issues using a family-focused approach to reduce the adverse effects on offspring mental health rather than the conventional gender-focused approach limited to maternal prenatal and postnatal mental health issues or individual treatment of the offspring.

## Teaser

This systematic review and meta-analysis examines the association of paternal depression with depression in offspring.

## Introduction

The neurodevelopmental theory of depression suggests that factors originating during earlier stages of human life are linked with an increased likelihood of depressive symptoms later in life.^1,2^ Among fathers, paternal depression is a risk factor potentially associated with increased risk of depression in their offspring and may be the consequence of the individual or combined influences of genetics and the developmental environment.^3,4,5,6,7,8,9^

The association between maternal depression and offspring depression is well investigated, with maternal depression identified as an important risk factor for offspring depression.^10,11^ While there is increasing awareness of the role that paternal depression can have in child development and later psychosocial outcomes, this topic has not been as thoroughly researched as the relationship between maternal mental health and that of their offspring. The available evidence shows inconsistent findings on the association between paternal depression and depression risk in offspring. While some studies identified an increased risk of depression in offspring exposed to paternal depression,^12,13,14^ other epidemiological studies have reported no associations.^15,16,17^ For example, a population-based study by Jacob et al^12^ revealed that the children of fathers with depressive disorders have a 61% increased risk of developing depression when compared with the children of fathers without such disorders. In a national comorbidity survey, Lies et al^18^ found that offspring with a depressed father was more than 2 times as likely to experience 12-month (OR, 2.03; 95% CI, 1.28-3.21) and lifetime depressive disorder (OR, 2.57; 95% CI, 1.87-3.54) when compared with offspring with no depressed father. A prospective, longitudinal community study by Lieb et al^19^ and a more recent birth cohort study by Liang et al^20^ reported up to a 3-fold increased risk of depression in offspring of depressed fathers (OR, 3.10; 95% CI, 2.0-4.80) and 2.64 (95% CI, 2.33-2.99), respectively.

Conversely, a prospective cohort study in Australia^15^ and a population-based cross-sectional survey in the Netherlands found no increased risk of depression in offspring exposed to paternal depression.^16^ Similarly, a UK-based pregnancy cohort study by Pearson et al^21^ that used data from the Avon Longitudinal Study of Parents and Children also found no association between paternal depression and increased risk of depression in young adult offspring. Given that paternal depression is reasonably common and fathers are increasingly involved in the care of their children, taking into account paternal depression might help to improve offspring mental health. Hence, we performed this systematic review and meta-analysis on the association between paternal and offspring subsequent depression to assess existing evidence and provide recommendations for future research to inform interventions.

## Methods

### Research Design

This systematic review and meta-analysis was performed following the Preferred Reporting Items for Systematic Review and Meta-analysis (PRISMA) reporting guideline. The review protocol was prospectively registered in the International Prospective Registration of Systematic Reviews (PROSPERO) and publicly available (CRD42020213983). All included studies received an ethical review, and all participants provided either verbal or written informed consent.

### Data Source and Searches

We conducted a comprehensive systematic search of the literature in 5 reputable electronic databases─Embase, PubMed, Scopus, PsycInfo, and Web of Science from database inception to December 15, 2022. Studies reporting the association between paternal depression and offspring depression have been searched without publication date restriction. Two independent investigators (G. A. and B. D.) screened the full-text articles for eligibility. The search was performed using the following relevant search terms: (*depression OR depressive OR psychopathology OR psychiatric disorder*) *AND* (*children OR offspring*) *AND* (*parental OR paternal OR father*) (eTable 1 in Supplement 1). The reference lists of the eligible studies were manually searched for additional studies.

### Study Selection and Eligibility Criteria

A study was included in this systematic review and meta-analysis if all the following criteria were met: (1) was based on humans; (2) written in the English language; (3) conducted using observational study design—case-control, cohort, and cross-sectional study designs; (4) examined the association between paternal depression and risk of depression in offspring; and (5) estimated the association using odds ratio (OR) or relative risks (RR) or reported data to calculate the effect estimates. Animal studies, reviews, case reports, commentaries, editorials, and meeting or conference abstracts were excluded from the current meta-analysis.

### Data Extraction and Study Quality

Two authors (G. A. and B. D.) extracted data independently using a standardized data extraction form and in accordance to PRISMA guidelines. Discrepancies were resolved through discussion. The methodologic quality of eligible articles was independently evaluated by 2 authors (G. A. and B. D.) using the Newcastle-Ottawa Scale (NOS) for observational studies^22,23^ (eMethods in Supplement 1).

### Data Synthesis and Analysis

The summary ORs and 95% CIs were pooled and a conventional meta-misanalysis was performed. Then, we conducted a cumulative meta-analysis to further determine how the pooled estimate changed over time (by year). For this, the eligible studies were first ordered by increasing year of publication, and then analysis was performed after sequential inclusion of 1 newly published study each time. This technique helps to enhance statistical power, determine how the pooled estimate and its precision changes over time, and explore potential sources of heterogeneity in the results. ORs were used as the measure of association for all studies. When studies provided effect estimates other than OR—including RR, Cohen *d*, mean differences, correlation coefficients (*r* or β-coefficients)—these were converted to OR. When a study reported data from multiple countries, each country’s data set was treated as an individual study. For studies that reported a number of models, effect estimates from the most adjusted model were used to minimize confounding bias and obtain a more precise estimate of the independent variable’s effect on the outcome. To account for the heterogeneity between the studies, summary ORs were pooled using inverse variance weighted random effect meta-analysis.

We used Cochrane *Q* and *I^2^* tests to evaluate the heterogeneity across the studies.^24^ The *I^2^* values of 25%, 50%, and 75% were considered low, moderate, and high heterogeneity, respectively.^25^ The presence of potential publication bias was examined statistically using the Egger test and graphically using a funnel plot.^26^

Subgroup analyses by the study design, timing of exposure, outcome diagnosis (depressive disorders or depressive symptoms), adjustment for confounders, and study quality were conducted. To investigate how each study affected overall effect size, we carried out a leave-one-out sensitivity analysis by excluding each study at a time and calculating the pooled effect estimates for the remaining studies. Stata version 16 (StataCorp LLC) was used to conduct the analysis. All statistical tests were conducted with 2-tailed statistical significance levels set at *P* < .05.

## Results

**Figure 1.**
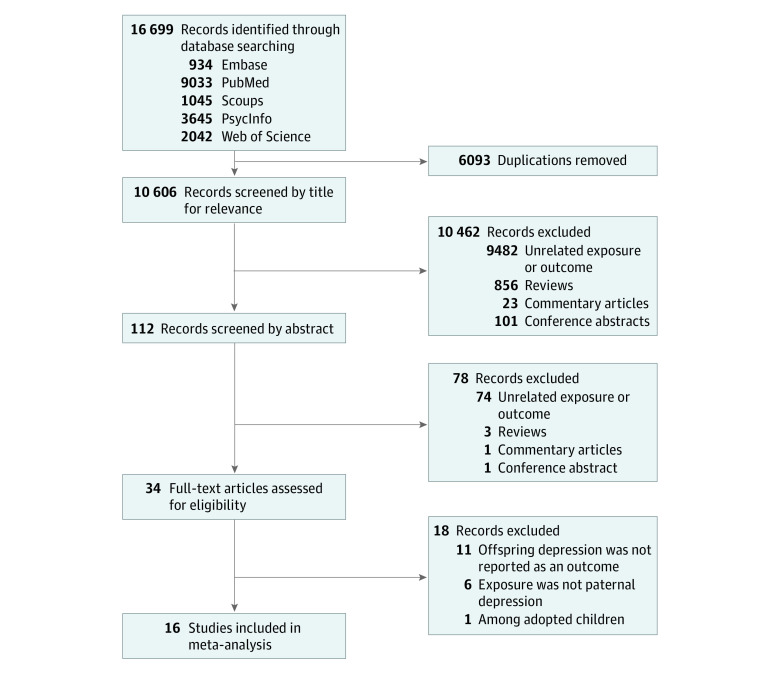
PRISMA Flow Chart of Included Studies

The initial systematic literature search yielded 16 699 citations. After removing duplicates from the initial pool (6093 citations), the remaining 10 606 records were screened by title and abstract for relevance, and 10 574 records were excluded. Finally, 34 full-text articles were reviewed, and 16 eligible studies were identified and included in this review. The manual search of the reference lists of included studies yielded no additional relevant studies (Figure 1).

### Characteristics of Included Studies

eTable 2 in Supplement 1 shows the characteristics of studies included in this systematic review and meta-analysis. The studies were first published between 2002^19,27^ and 2021.^20^ All studies, except 1,^20^ were conducted in high-income countries—8 in Europe,^13,14,16,17,19,21,28^ 6 in the US,^3,12,18,29,30,31^ and 1 in Australia.^15^ Of the included studies, 14 were cohort, and 2 were cross-sectional. The sample size of the studies included in this systematic review and meta-analysis ranged from 220 to 4 138 151 participants.^12,20^

### Confounding Adjustment

Of the 16 included studies, 14 provided adjusted effect estimates. Parental age and offspring gender were the most confounder factors accounted for by most studies. While 4 studies adjusted for maternal depression and substance use, 2 studies adjusted for other paternal psychiatric disorders. Likewise, other important confounders, such as maternal and paternal education, family income, marital status, and pregnancy complications, were not consistently accounted for in the original study results (Table 1).

**Table 1.** Confounder Variables Accounted by Studies Included in the Systematic Review and Meta-Analysis

Study	Confounders adjustments
Brennan et al,^27^ 2002	Mother’s education, child’s gender, and family income
Lieb et al,^19^ 2002	Age and gender of the offspring
Klein et al,^3^ 2005	Offspring gender, parental education, maternal major depressive disorder, maternal anxiety disorder, and maternal substance abuse disorder, paternal anxiety disorder, and paternal substance abuse disorder
Rohde et al,^30^ 2005	Maternal depression and whether the offspring is living with parent
Ramchandani et al,^17^ 2008	Maternal depression and parental education status
Reeb et al,^31^ 2010	Offspring gender, family functioning, economic hardship, and maternal depressive symptoms
Lies et al,^18^ 2010	Offspring gender, race, education, and employment status
Pearson et al,^21^ 2013	No information provided
Reeb et al,^29^ 2015	Maternal depression and parental education status
Jacobs et al,^12^ 2015	Age, parental comorbidity (substance use and anxiety disorders) and parental living arrangement (present in the home)
Musliner et al,^14^ 2015	Offspring gender, age, calendar time, place of birth, mothers age at birth, father’s age at birth, mother’s and father’s hierarchical psychiatric diagnoses variables
Middeldorp et al,^16^ 2016	No adjustment made
Lewis et al,^28^ 2017	Family income; paternal and maternal education; paternal, maternal, and child’s age at time of exposure; gender of child; ethnicity; whether father is biological parent; and paternal and maternal alcohol use at time of exposure, child emotional symptoms at time of exposure, paternal and maternal reports of interparental relationship conflict
Gutierrez-Galve et al,^13^ 2019	Paternal education and paternal age
Liang et al,^20^ 2021	Parental age at childbirth, gender of children, income, and residence

### Quality Assessment

Among the 14 cohort studies included in this meta-analysis, 12 were classed as high in quality^3,12,13,14,17,19,20,28,29,30,31^ and 2 as moderate quality^21,27^ based on the NOS tool. While six studies scored 9, another six scored 8, and two studies scored 7. Both cross-sectional studies included in this meta-analysis were good quality studies^16,18^ (eTable 3 in Supplement 1).

### Meta-Analysis

**Figure 2.**
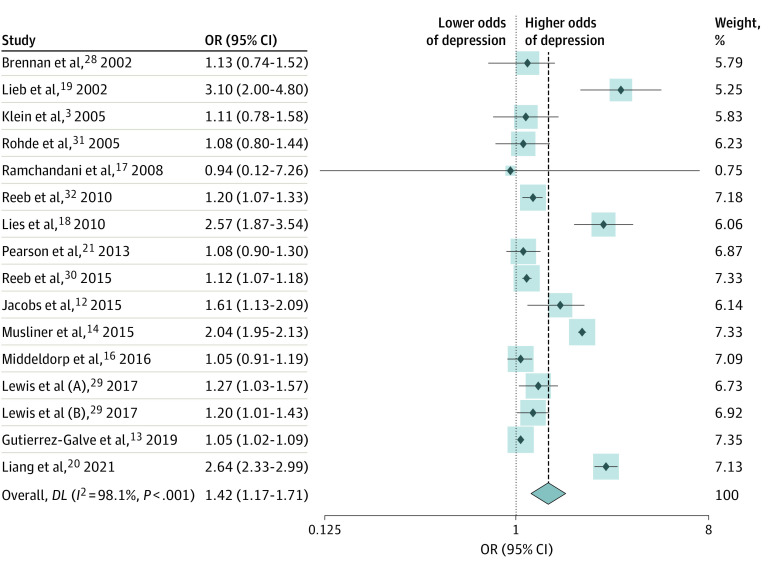
Forest Plot for Conventional Cumulative Random-Effect Meta-Analysis Boxes indicate weight of studies in meta-analysis.

**Figure 3.**
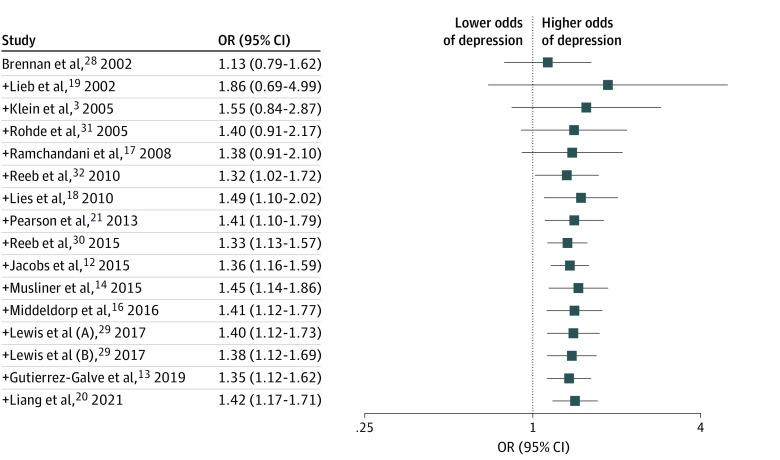
Forest Plot for Cumulative Random-Effect Meta-Analysis The plus symbol indicates sequential addition of the study results to those previously published.

Eleven of the 16 included studies reported a significant positive association between paternal depression and increased risk of depression in their children.^3,12,13,14,18,19,20,28,29^ A random effect meta-analysis of included studies indicated that the risk of depression was 42% higher in offspring exposed to paternal depression than those not exposed to paternal depression (OR, 1.42; 95% CI, 1.17-1.71) (Figure 2). A cumulative random-effect meta-analysis model performed after sequential inclusion of a newly published study one at a time also provided consistent findings (OR, 1.42; 95% CI, 1.17-1.71). Observed estimates reported by studies published after Lies et al^18^ in 2010 study did not change in the direction of association despite resulting in minimal changes to the point estimates (OR) and precision (95% CI) (Figure 3).

### Subgroup and Sensitivity Analysis

Our stratified analysis revealed that the risk of offspring depression was considerably higher in studies that involved paternal depressive disorders as exposure (OR, 1.65; 95% CI, 1.28-2.12) compared with those studies that used paternal depressive symptoms as exposure (OR, 1.12; 95% CI, 1.06-1.19). Similarly, the effect size of the association was higher for offspring exposed to lifetime paternal depression (OR, 1.58; 95% CI, 1.09-2.29) when compared with those exposed to paternal depression during postpartum (OR, 1.05; 95% CI, 1.02-1.09) and early childhood periods (OR, 1.22; 95% CI, 1.07-1.36). Two of the included studies reported only unadjusted effects estimates. The associations remained unchanged when we excluded them from the main results (OR, 1.47; 95% CI, 1.24-1.74). The risk of offspring depression was slightly higher in studies that did not account for the effects of maternal alcohol and/or other drug use during prenatal and perinatal periods (OR, 1.46; 95% CI, 1.17-1.83) as compared with studies that did account for such exposures (OR, 1.26; 95% CI, 1.12-1.43). Likewise, effect sizes were higher for studies that did not account for maternal depression (OR, 1.51; 95% CI, 1.19-1.93) than in studies that accounted for maternal depression (OR, 1.12; 95% CI, 1.07-1.17).

We also conducted a subgroup analysis based on the scales used to measure depression in offspring and found a considerably elevated risk of depression in studies that used diagnostic instruments (ie, assessed depressive disorders) (OR, 1.56; 95% CI, 1.17-2.07) when compared with studies that used screening instrument (ie, assessed depressive symptoms) (OR, 1.14; 95% CI, 1.09-1.19). In studies that used a cohort design, paternal depression was associated with a 39% increased risk of depression in offspring (OR, 1.39; 95% CI, 1.14-1.70). We found no evidence of associations among cross-sectional studies (OR, 1.62; 95% CI, 0.68-3.90) (eTable 4 in Supplement 1).

Table 2 shows the results of leave-one-out analyses. When one of the study estimates was iteratively removed from the analysis, the pooled effect estimates did not change substantively, varying between 1.35 (95% CI, 1.12-1.62) and 1.45 (95% CI, 1.18-1.78).

**Table 2.** Leave-One-Out Sensitivity Analysis

Study omitted	OR (95% CI)
Brennan et al,^27^ 2002	1.44 (1.18-1.74)
Lieb et al,^19^ 2002	1.36 (1.12-1.64)
Klein et al,^3^ 2005	1.44 (1.18-1.74)
Rohde et al,^30^ 2005	1.44 (1.19-1.75)
Ramchandani et al,^17^ 2008	1.42 (1.17-1.71)
Reeb et al,^31^ 2010	1.43 (1.17-1.75)
Lies et al,^18^ 2010	1.36 (1.12-1.64)
Pearson et al,^21^ 2013	1.44 (1.18-1.76)
Reeb et al,^29^ 2015	1.44 (1.16-1.80)
Jacobs et al,^12^ 2015	1.40 (1.16-1.70)
Musliner et al,^14^ 2015	1.36 (1.18-1.57)
Middeldorp et al,^16^ 2016	1.45 (1.19-1.76)
Lewis et al,^28^ 2017 (Ireland)^a^	1.43 (1.17-1.73)
Lewis et al,^28^ 2017 (UK)^a^	1.43 (1.18-1.74)
Gutierrez-Galve et al,^13^ 2019	1.45 (1.18-1.78)
Liang et al,^20^ 2021	1.35 (1.12-1.62)
Combined	1.42 (1.17-1.71)

^a^ This study included 2 populations based in Ireland and the UK.

### Assessment Publication Bias and Variability Between Studies

There was symmetry in the funnel plot for studies on the association between paternal depression and risk of offspring depression, and our quantitative test using Egger test demonstrated no evidence of substantial publication bias (β [SE], 2.36 [4.0]; *P* = .57) (eFigure in Supplement 1). We observed substantial heterogeneity among studies included in the meta-analysis (*I^2^* = 98.1%; *Q* = 772.9; *P* < .001).

## Discussion

To our knowledge, this is the first systematic review and meta-analysis to investigate whether paternal depression is associated with an increased risk of offspring depression. We found a 42% higher risk of depression in offspring exposed to paternal depression. The association between paternal depression and depression risk in offspring remained consistent since Lies et al’s study in 2010,^18^ suggesting that the evidence for a statistical association was sufficient since that time. We noted a considerably higher risk of depression in offspring exposed to paternal depressive disorders than those exposed to depression as defined by a nonclinical symptom scale. The observed association did not alter by the adjustment to confounders, including maternal depression, maternal prenatal and perinatal substance use, parental age, family income, parental education, and comorbid other paternal psychiatric disorders. The association between maternal depression and offspring depression risk is well investigated, with maternal depression identified as an important risk factor for offspring depression.^32,33,34^ Hence, findings from this meta-analysis suggest the importance of addressing paternal mental health issues (ie, family-focused approach) to reduce the adverse effects on the mental health of offspring, rather than the traditional gender-focused approach limited to potential impact of maternal prenatal and perinatal mental health issues.^35^

The mechanisms that underlie the association between paternal and offspring depression remain unclear. Genetic and epigenetic mechanisms are most frequently reported as potential explanations for the increased risk of depression in children with paternal depression.^5,6,8,9^ In support of this explanation, a 2010 study that explored the pathophysiology of depression revealed that genetics are responsible for 40% of the risk for depression.^5^ Other studies examining the etiologic sources of intergenerational transmission of depression have also reported consistent findings.^6,36,37^ Epigenetic mechanisms could also contribute to the transmission of paternal depression across multiple generations, often occurring through DNA methylation and histone modifications, which play an important role in the regulation of cellular functions by mediating communication between the genome and the environment.^8,38,39,40^ Existing evidence suggests that DNA methylation and histone modifications can reliably differentiate the stable and heritable characteristics of depression.^9,41^

The association between parental depression and subsequent depression in offspring has also been explained, in part, by how depression may affect parental sensitivity toward the child, the security of the attachment and parenting, which might, in turn, increase the risk of depression in offspring.^42,43^ Paternal depression has also been associated with an increased risk of psychoactive substance use, including alcohol and drugs.^7^ Parental substance use and related undesirable changes in family situations, including negative parent-child interactions and divorce or separations,^44,45^ could negatively impact the cognitive and psychological development of offspring and later increase the likelihood of depression.^45,46,47,48^

However, it should be borne in mind that the observed associations between paternal depression and offspring depression risk could also be due to the confounding effects of unmeasured variables. While 3 of the studies have reported unadjusted estimates, the remaining studies did not consistently account for the most important confounders, including maternal and paternal depression and substance use, parental age, parental medical conditions, and other sociodemographic variables, as well as child-related factors, including adverse childhood experiences and other comorbid psychiatric problems. Emerging epidemiological data showed that these factors are associated with an increased risk of depression.^2,49^ However, our subgroup analysis by confounder adjustment provided consistent findings, suggesting the robustness of the associations seen in this meta-analysis.

### Strengths and Limitations

This systematic review and meta-analysis had several strengths. To our knowledge, this study is the first to comprehensively examine the association between paternal depression and depression risk in offspring. The cumulative meta-analysis method provided additional information on how the pooled estimate and its precision changed over time as each newly published study was added to the pool, unlike the traditional meta-analysis method. We conducted a subgroup and sensitivity analysis to explore the source of heterogeneity of included studies. We separately estimated depression risk in offspring exposed to paternal depressive disorders and elevated paternal depressive symptoms compared with unexposed counterparts. Finally, data extraction and methodological quality assessment of included studies were conducted by 2 reviewers independently, and most of the included studies were high in quality according to the NOS score.

Our study had some limitations. Most included studies did not consistently account for relevant confounders such as maternal depression, and some reported unadjusted effect estimates, which may have influenced the meta-analytic results. The noncollapsibility nature of OR also needs to be taken into account when interpreting the findings of the current meta-analysis. We were also unable to examine whether the observed associations were gender sensitive. As we restricted our search to English articles only, relevant studies published in a language other than English may have been missed. Unpublished research and gray literature was also not included, possibly resulting in publication bias. However, tests for publication bias demonstrated no asymmetry based on funnel plot, and the Egger test also confirmed nonsignificant bias coefficients, suggesting the absence of publication bias. Finally, as all studies except Liang et al^20^ were conducted in developed countries, the results may not be generalizable to other populations in developing countries.

## Conclusions

This meta-analysis of observational studies showed that paternal depression was significantly associated with greater depression risk in offspring. Offspring exposed to paternal depressive disorders had a higher risk of later depression than those exposed to depressive symptoms. These findings suggest the importance of addressing maternal and paternal mental health issues using a family-focused approach to reduce the adverse effects on offspring mental health and cognitive development rather than the conventional gender-focused approach limited to maternal prenatal and postnatal mental health issues or individual treatment of the offspring. The potential underlying mechanisms linking paternal depression with offspring depression warrant further studies.

## Notes

Supplement 1. **eMethods.** Data Extraction and Study Quality **eFigure.** Funnel Plot for Assessing Potential Publication Bias **eTable 1.** Search Terms and Strategy **eTable 2.** Characteristics of Included Studies **eTable 3.** Quality Assessment of Studies Included in the Final Analysis According to the Newcastle-Ottawa Scale (NOS) **eTable 4.** Summary of the Subgroup Analysis
eReferences.

Supplement 2.
Data Sharing Statement
