# Supplementary material for: Paternal Depression and Risk of Depression Among Offspring: A Systematic Review and Meta-Analysis
Source: JAMA Netw Open. 2023 Aug 16;6(8):e2329159. doi: 10.1001/jamanetworkopen.2023.29159 (PMC10433087; doi:10.1001/jamanetworkopen.2023.29159)
Supplement: Supplement 1. — eMethods. Data Extraction and Study Quality eFigure. Funnel Plot for Assessing Potential Publication Bias eTable 1. Search Terms and Strategy eTable 2. Characteristics of Included Studies eTable 3. Quality Assessment of Studies Included in the Final Analysis According to the Newcastle-Ottawa Scale (NOS) eTable 4. Summary of the Subgroup Analysis eReferences. [file jamanetwopen-e2329159-s001.pdf]

Supplemental Online Content

Dachew B, Ayano G, Duko B, Lawrence B, Betts K, Alati R. Paternal depression and risk of depression among offspring: a systematic review and meta-analysis. *JAMA Netw Open.* 2023;6(8):e2329159.
doi:10.1001/jamanetworkopen.2023.29159

eMethods. Data Extraction and Study Quality

eFigure. Funnel Plot for Assessing Potential Publication Bias

eTable 1. Search Terms and Strategy

eTable 2. Characteristics of Included Studies

eTable 3. Quality Assessment of Studies Included in the Final Analysis According to the Newcastle-Ottawa Scale (NOS)

eTable 4. Summary of the Subgroup Analysis

eReferences.

This supplemental material has been provided by the authors to give readers additional information about their work.

© 2023 Dachew B et al. *JAMA Network Open.*

eMethods

Data extraction

Two authors (GA and BD) extracted data and independently using a standardised data extraction form. For each eligible study, we extracted the following information: first author's name, year of publication, geographic location, study design, the timing of exposure and outcomes assessment, an instrument used to assess cases in exposed and control groups, sample size, confounders adjusted for, the effects estimates (OR/RR with 95% confidence interval).

Study quality

Two investigators (GA and BD) independently evaluated the methodologic quality of the eligible articles using the Newcastle-Ottawa Scale (NOS) for observational studies [1], with disagreements resolved by discussion. The scoring standard in the NOS scale was mainly based on three broad domains: selection of the study groups, ascertainment of outcome and exposure variables, and comparability between the groups. For cohort studies, the scoring was grouped into three categories: low quality (scored 0-3), moderate quality (scored 4-6) and high quality (scored 7-9). The maximum possible score is nine, which represents the highest methodological quality. The adapted version of NOS was used for cross-sectional studies, and scores were classified into four categories— very good quality (scored 9-10), good quality (scored 7-8), satisfactory quality (scored 5-6), and unsatisfactory (0-4 points) [2].

© 2023 Dachew B et al. *JAMA Network Open.*

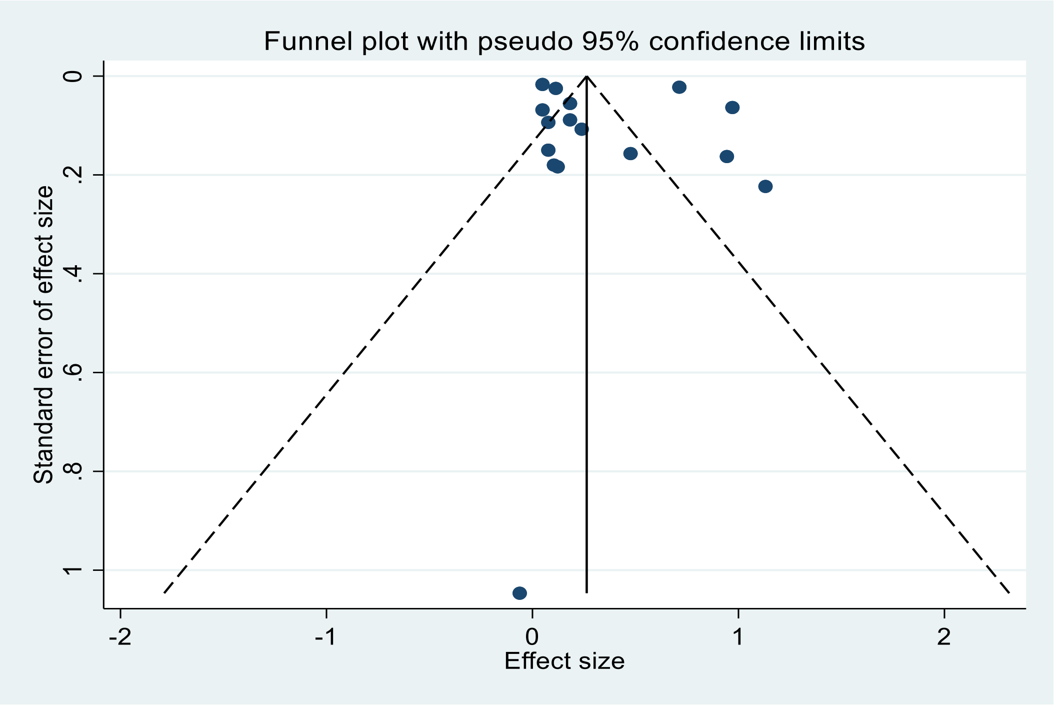

eFigure. Funnel plot for assessing potential publication bias

© 2023 Dachew B et al. *JAMA Network Open.*

eTable 1. Example search terms and strategy (PubMed)

**Search:** ((depression OR depressive OR psychopathology OR psychiatric disorder) AND (children OR offspring)) AND (paternal or father) ("depressed"[All Fields] OR "depression"[MeSH Terms] OR "depression"[All Fields] OR "depressions"[All Fields] OR "depression s"[All Fields] OR "depressive disorder"[MeSH Terms] OR ("depressive"[All Fields] AND "disorder"[All Fields]) OR "depressive disorder"[All Fields] OR "depressivity"[All Fields] OR "depressive"[All Fields] OR "depressively"[All Fields] OR "depressiveness"[All Fields] OR "depressives"[All Fields] OR ("depressed"[All Fields] OR "depression"[MeSH Terms] OR "depression"[All Fields] OR "depressions"[All Fields] OR "depression s"[All Fields] OR "depressive disorder"[MeSH Terms] OR ("depressive"[All Fields] AND "disorder"[All Fields]) OR "depressive disorder"[All Fields] OR "depressivity"[All Fields] OR "depressive"[All Fields] OR "depressively"[All Fields] OR "depressiveness"[All Fields] OR "depressives"[All Fields]) OR ("psychopathologies"[All Fields] OR "psychopathology"[MeSH Terms] OR "psychopathology"[All Fields]) OR ("mental disorders"[MeSH Terms] OR ("mental"[All Fields] AND "disorders"[All Fields]) OR "mental disorders"[All Fields] OR ("psychiatric"[All Fields] AND "disorder"[All Fields]) OR "psychiatric disorder"[All Fields])) AND ("child"[MeSH Terms] OR "child"[All Fields] OR "children"[All Fields] OR "child s"[All Fields] OR "children s"[All Fields] OR "childrens"[All Fields] OR "childs"[All Fields] OR ("offspring"[All Fields] OR "offspring s"[All Fields] OR "offsprings"[All Fields])) AND ("paternal"[All Fields] OR "paternally"[All Fields] OR "paternity"[MeSH Terms] OR "paternity"[All Fields] OR "paternities"[All Fields] OR ("father s"[All Fields] OR "fathered"[All Fields] OR "fathers"[MeSH Terms] OR "fathers"[All Fields] OR "father"[All Fields] OR "fathering"[All Fields]))

© 2023 Dachew B et al. *JAMA Network Open.*

eTable 2: Characteristics of included studies

Author (year)	Country	Sample size	Effect estimate in OR (95% CI)	Study Design	Exposure measures	Outcome measures	Timing of parental depression diagnosis	Child age at outcome measure
Brennan et al.,[3] 2002	Australia	522	1.13 (0.74 – 1.52)	Cohort	SCID (DSM–IV)	DSM–IV (K-SADS-E	Lifetime	15 years
Lieb et al.,[4] 2002	Germany	2,427	3.10 (2.00 – 4.80)	Cohort	DSM-IV	DSM-IV (M-CIDI)	14-17 years postpartum	17-21years
Klein et al.,[5] 2005	USA	775	1.11 (0.78 – 1.58)	Cohort	DSM-IV	DSM-III R	Lifetime	14- 24 years
Rohde et al.,[6] 2005	USA	244	1.08 (0.80 – 1.44)	Cohort	DSMIV, (SCID-NP)	DSM-III-R (K-SADS)	19 years postpartum	24 years
Ramchandani et al.,[7] 2008	UK	10,975	0.94 (0.12 – 7.26)	Cohort	EPDS	DAWBA	18 moths antenatal and 8 weeks, 8 months and 21 months postpartum	7 years
Reeb et al.,[8] 2010	USA	451	1.20 (1.07 – 1.33)	Cohort	SCL-90-R	SCL-90-R	13.2 years postpartum	13.2 years
Lies et al.,[9] 2010	USA	1,255	2.57 (1.87 – 3.54)	Cross-sectional	DSM-III R (FH-RDC)	DSM-III	Lifetime	18 to 29 years
Pearson et al.,[10] 2013	UK	4,500	1.08 (0.90 – 1.30)	Cohort	EPDS	CIS-R (ICD-10)	18 and 32 weeks antenatally	18 years
Reeb et al.,[11] 2015	USA	395	1.12 (1.07 – 1.18)	Cohort	SCL-90-R	SCL-90-R	12.6 years postpartum	20-22 years
Jacobs et al.,[12] 2015		220	1.61 (1.13 – 2.09)	Cohort	KSADS-PL / SADS-LA)	DSM-IV	Lifetime	6-23yrs
Musliner et al.,[13] 2015	Denmark	2,976,264	2.04 (1.95 – 2.13)	Cohort	ICD-8/10	ICD-10	Lifetime	30.5-33.6 years
Middeldorp et.al.,[14] 2016	Netherlands	530	1.05 (0.91 – 1.19)	Cross-sectional	CBCL and ASR	CBCL and ASR	Lifetime	7-15 years

© 2023 Dachew B et al. *JAMA Network Open.*

Lewis et al.,[15] 2017 (a)	Ireland	6,070	1.27 (1.03 – 1.57)	Cohort	K6+	SMFQ	9 years postpartum	13 years
Lewis et al., [15]2017 (b)	UK	7,768	1.20 (1.01 – 1.43)	Cohort	CES-D	SMFQ	7 years postpartum	14 years
Gutierrez-Galve et al.,[16] 2019	UK	3,176	1.05 (1.02 – 1.09)	Cohort	EPDS	CIS-R (ICD-10)	8 weeks and 8 months postnatally	18 years
Liang et al.,[17] 2021	Taiwan	4,138,151	2.64 (2.33 –2.99)	Cohort	ICD-9-CM	ICD-9-CM	At childbirth	7-20 years

Adult self-report (ASR); Affective Disorders and Schizophrenia for School-Age Children (K-SADS); Centre for Epidemiological Studies Depression Scale (CES-D); Child Behaviour Checklist (CBCL); Clinical Interview Schedule-Revised (CIS-R); Development and Well-Being Assessment (DAWBA); Diagnostic and Statistical Manual of Mental Disorders (DSM); Diagnostic and Statistical Manual of Mental Disorders-Revised (DSM-III-R); Edinburgh Postnatal Depression Scale (EPDS); Family History - Research Diagnostic Criteria (FH-RDC); International Classification of Diseases (ICD); International Classification of Diseases, Ninth Revision, Clinical Modification (ICD-9-CM); Kessler six-item psychological distress scale (K6+); Kiddie-Schedule for Affective Disorders and Schizophrenia Epidemiologic or Present and Lifetime version (KSADS-PL); Munich-Composite International Diagnostic Interview (M-CIDI); Schedule for Affective Disorders and Schizophrenia Lifetime version (SADS-LA); Short Mood and Feelings Questionnaire (SMFQ); Structured Clinical Interview for DSM-IV (SCID); Structured Clinical Interview for DSM-IV, non-patient version (SCID-NP); and Symptom Checklist-90-Revised (SCL-90-R).

© 2023 Dachew B et al. *JAMA Network Open.*

eTable 3. Quality assessment of studies included in the final analysis according to the Newcastle-Ottawa scale (NOS)

Study name	Selection	Comparability	Exposure/outcome	Total	Overall quality
**Cohort studies**					
Reeb et al., 2010	4	1	3	8	High
Reeb et al., 2015	4	2	3	9	High
Brennan et al., 2002	3	1	3	7	Moderate
Jacobs et al., 2015	4	2	3	9	High
Lieb et al., 2002	4	1	3	8	High
Musliner et al., 2015	4	2	3	9	High
Gutierrez-Galve et al., 2019	4	1	3	8	High
Pearson et al., 2013	3	1	3	7	Moderate
Lewis et al., 2017 (a)	4	2	3	9	High
Lewis et al., 2017 (b)	4	2	3	9	High
Klein et al. 2005	4	2	3	9	High
Ramchandani et al., 2008	4	1	3	8	High
Rohde et al., 2005	4	1	3	8	High
Liang et al ., 2021	4	1	3	8	High
**Cross-sectional studies**					
Lies et al., 2010	4	1	3	8	Good
Middeldorp et.al., 2016	4	1	3	8	Good

Key: For Cohort studies High quality: NOS score above or equal to 8; Moderate quality: NOS score of 6 and 7 and Low quality: NOS score below 6

For cross-sectional studies: very good quality (scored 9-10), good quality (scored 7-8), satisfactory quality (scored 5-6), and unsatisfactory studies (0-4 points)

© 2023 Dachew B et al. *JAMA Network Open.*

eTable 4. Summary of the subgroup analysis

Subgroups	OR	95% CI	Heterogeneity across the studies		Heterogeneity between the groups (P-value)
			I2	P-value	
**Study design**					.74
Cohort (n = 14)	1.39	1.14-1.70	98.3	<.001	
Cross-sectional (n = 2)	1.62	0.68-3.90	96.1	<.001	
**Study Quality**					.66
High (n = 12)	1.45	1.16-1.81	98.5	<.001	
Good/moderate (n = 4)	1.32	0.95-1.84	88.7	<.001	
**Tool used to assess outcome**					.03
Screening (n = 5)	1.14	1.09-1.19	0.00	0.61	
Diagnostic (n = 11)	1.56	1.17-2.07	98.6	<.001	
**Period outcome assessed**					.21
Childhood/adolescence (n = 7)	1.33	0.97-1.84	0.00	0.64	
Adulthood (n = 6)	1.38	1.02-1.86	99.2	<.001	
Both (n = 3)	1.74	1.03-2.96	99.3	<.001	
**Tool used to assess exposure**					.003
Screening (n =6)	1.12	1.06-1.19	58.7	.03	
Diagnostic (n =10)	1.65	1.28-2.12	98.6	<.001	
**Exposure time**					.009
Childhood (n = 4)	1.22	1.07-1.36	0.00	0.84	
Adolescence (n = 3)	1.38	1.09-1.74	90.6	<.001	
Lifetime (n = 5)	1.58	1.09-2.29	95.9	<.001	
Postpartum (n = 3)	1.05	1.02-1.09	0.00	0.95	
Adulthood (n = 1)	1.08	0.80-1.42	-	-	
**Adjusted for any confounders**					.004
Yes (n = 13)	1.51	1.22-1.87	98.4	<.001	
No (n = 3)	1.07	0.96-1.18	0.00	0.92	
**Adjusted for maternal substance use**					.26
Yes (n = 4)	1.26	1.12-1.43	7.5	0.36	
No (n =12)	1.46	1.17-1.83	98.6	<.001	
**Adjusted for maternal depression**					.02
Yes (n = 4)	1.12	1.07-1.17	0.0	0.99	
No (n =12)	1.51	1.19-1.93	98.5	<.001	
**Adjusted for paternal mental disorders**					.80
Yes (n = 2)	1.35	0.94-1.94	58.8	0.12	
No (n = 14)	1.43	1.17-1.74	98.1	<.001	

© 2023 Dachew B et al. *JAMA Network Open.*

eReferences

1. Wells G. Wells GA, Shea B, O'Connell D, Peterson J, Welch V, Losos M, et al. The Newcastle-Ottawa Scale (NOS) for assessing the quality of nonrandomised studies in meta-analyses. In:2011.
2. Modesti PA, Reboldi G, Cappuccio FP, et al. Panethnic Differences in Blood Pressure in Europe: A Systematic Review and Meta-Analysis. *PLoS One.* 2016;11(1):e0147601.
3. Brennan PA, Hammen C, Katz AR, Le Brocque RM. Maternal depression, paternal psychopathology, and adolescent diagnostic outcomes. 2002;70:1075-1085.
4. Lieb R, Isensee B, Höfler M, Pfister H, Wittchen H-U. Parental Major Depression and the Risk of Depression and Other Mental Disorders in Offspring: A Prospective-Longitudinal Community Study. *Archives of General Psychiatry.* 2002;59(4):365-374.
5. Klein DN, Lewinsohn PM, Rohde P, Seeley JR, Olino TM. Psychopathology in the adolescent and young adult offspring of a community sample of mothers and fathers with major depression. *Psychol Med.* 2005;35(3):353-365.
6. Rohde P, Lewinsohn PM, Klein DN, Seeley JR. Association of parental depression with psychiatric course from adolescence to young adulthood among formerly depressed individuals. *J Abnorm Psychol.* 2005;114(3):409-420.
7. Ramchandani PG, Stein A, O'Connor TG, Heron J, Murray L, Evans J. Depression in men in the postnatal period and later child psychopathology: a population cohort study. *J Am Acad Child Adolesc Psychiatry.* 2008;47(4):390-398.
8. Reeb BT, Conger KJ, Wu EY. Paternal Depressive Symptoms and Adolescent Functioning: The Moderating Effect of Gender and Father Hostility. *Fathering.* 2010;8(1):131-142.
9. Leis JA, Mendelson T. Intergenerational transmission of psychopathology: minor versus major parental depression. *J Nerv Ment Dis.* 2010;198(5):356-361.
10. Pearson RM, Evans J, Kounali D, et al. Maternal depression during pregnancy and the postnatal period: risks and possible mechanisms for offspring depression at age 18 years. *JAMA Psychiatry.* 2013;70(12):1312-1319.
11. Reeb BT, Wu EY, Martin MJ, Gelardi KL, Shirley Chan SY, Conger KJ. Long-term Effects of Fathers' Depressed Mood on Youth Internalizing Symptoms in Early Adulthood. *J Res Adolesc.* 2015;25(1):151-162.
12. Jacobs RH, Talati A, Wickramaratne P, Warner V. The Influence of Paternal and Maternal Major Depressive Disorder on Offspring Psychiatric Disorders. *J Child Fam Stud.* 2015;24(8):2345-2351.
13. Musliner KL, Trabjerg BB, Waltoft BL, et al. Parental history of psychiatric diagnoses and unipolar depression: a Danish National Register-based cohort study. *Psychol Med.* 2015;45(13):2781-2791.
14. Middeldorp CM, Wesseldijk LW, Hudziak JJ, Verhulst FC, Lindauer RJ, Dieleman GC. Parents of children with psychopathology: psychiatric problems and the association with their child's problems. *Eur Child Adolesc Psychiatry.* 2016;25(8):919-927.
15. Lewis G, Neary M, Polek E, Flouri E, Lewis G. The association between paternal and adolescent depressive symptoms: evidence from two population-based cohorts. *The Lancet Psychiatry.* 2017;4(12):920-926.
16. Gutierrez-Galve L, Stein A, Hanington L, et al. Association of Maternal and Paternal Depression in the Postnatal Period With Offspring Depression at Age 18 Years. *JAMA Psychiatry.* 2019;76(3):290-296.
17. Liang CS, Bai YM, Hsu JW, et al. Associations of parental mental disorders and age with childhood mental disorders: a population-based cohort study with four million offspring. *Eur Child Adolesc Psychiatry.* 2021.

© 2023 Dachew B et al. *JAMA Network Open.*
