# Supplementary material for: Paternal Depression and Risk of Depression Among Offspring: A Systematic Review and Meta-Analysis
Source: JAMA Netw Open. 2023 Aug 16;6(8):e2329159. doi: 10.1001/jamanetworkopen.2023.29159 (PMC10433087; doi:10.1001/jamanetworkopen.2023.29159)
Supplement: Supplement 2. — Data Sharing Statement [file jamanetwopen-e2329159-s002.pdf]

# Data Sharing Statement

Dachew. Paternal Depression and Risk of Depression Among Offspring. *JAMA Netw Open*. Published August 16, 2023. doi:10.1001/jamanetworkopen.2023.29159

**Data**
**Data available:** No

**Additional Information**
**Explanation for why data not available:** No participant data was collected for this manuscript. All data will be available within the manuscript and has been collected by the respective authors of papers included in the meta-analysis.
